# Use of quantitative *in vitro* to *in vivo* extrapolation (QIVIVE) for the assessment of non-combustible next-generation product aerosols

**DOI:** 10.3389/ftox.2024.1373325

**Published:** 2024-04-11

**Authors:** Marjory Moreau, Liam Simms, Melvin E. Andersen, Edgar Trelles Sticken, Roman Wieczorek, Sarah Jean Pour, Fiona Chapman, Karin Roewer, Sandra Otte, Jeffrey Fisher, Matthew Stevenson

**Affiliations:** ^1^ Scitovation LLC, Durham, NC, United States; ^2^ Imperial Brands PLC, Bristol, United Kingdom; ^3^ Reemtsma Cigarettenfabriken GmbH, An Imperial Brands PLC Company, Hamburg, Germany

**Keywords:** physiologically based pharmacokinetic (PBPK) modelling, respiratory toxicity, *in vitro*, aerosol, heated tobacco product (HTP), cigarette, multiple-path particle dosimetry

## Abstract

With the use of *in vitro* new approach methodologies (NAMs) for the assessment of non-combustible next-generation nicotine delivery products, new extrapolation methods will also be required to interpret and contextualize the physiological relevance of these results. Quantitative *in vitro* to *in vivo* extrapolation (QIVIVE) can translate *in vitro* concentrations into in-life exposures with physiologically-based pharmacokinetic (PBPK) modelling and provide estimates of the likelihood of harmful effects from expected exposures. A major challenge for evaluating inhalation toxicology is an accurate assessment of the delivered dose to the surface of the cells and the internalized dose. To estimate this, we ran the multiple-path particle dosimetry (MPPD) model to characterize particle deposition in the respiratory tract and developed a PBPK model for nicotine that was validated with human clinical trial data for cigarettes. Finally, we estimated a Human Equivalent Concentration (HEC) and predicted plasma concentrations based on the minimum effective concentration (MEC) derived after acute exposure of BEAS-2B cells to cigarette smoke (1R6F), or heated tobacco product (HTP) aerosol at the air liquid interface (ALI). The MPPD-PBPK model predicted the *in vivo* data from clinical studies within a factor of two, indicating good agreement as noted by WHO International Programme on Chemical Safety (2010) guidance. We then used QIVIVE to derive the exposure concentration (HEC) that matched the estimated *in vitro* deposition point of departure (POD) (MEC cigarette = 0.38 puffs or 11.6 µg nicotine, HTP = 22.9 puffs or 125.6 µg nicotine) and subsequently derived the equivalent human plasma concentrations. Results indicate that for the 1R6F cigarette, inhaling 1/6th of a stick would be required to induce the same effects observed *in vitro*, *in vivo*. Whereas, for HTP it would be necessary to consume 3 sticks simultaneously to induce *in vivo* the effects observed *in vitro*. This data further demonstrates the reduced physiological potency potential of HTP aerosol compared to cigarette smoke. The QIVIVE approach demonstrates great promise in assisting human health risk assessments, however, further optimization and standardization are required for the substantiation of a meaningful contribution to tobacco harm reduction by alternative nicotine delivery products.

## 1 Introduction

Heated tobacco products (HTPs) offer an alternative to consuming nicotine without the need to burn tobacco or produce smoke. A portion of refined tobacco is heated in a controlled manner, never burnt, producing an inhalable aerosol which contains nicotine and flavor aromas from the tobacco. As HTPs eliminate the need to burn tobacco, and do not produce smoke, the aerosols they produce contain fewer and lower levels of harmful chemicals compared to cigarette smoke (Chapman et al., 2023). As such, heated tobacco products have the potential to provide a meaningful contribution to tobacco harm reduction (COT, 2017).

Traditionally, inhalation toxicity testing has been performed using animal models to identify various physiological outcomes, including the lethal concentration of airborne materials or maximum tolerable concentration. *In vitro* studies in which a solution of the aerosol ingredients is directly applied to cells in culture are not representative of an *in vivo* inhalation exposure due to a lack of gas phase exposure (Mulhopt et al., 2020). Additionally, these submerged *in vitro* conditions neither reflect realistic cell-cell communication within organ systems, cell-particle interactions and particle deposition characteristics as occurs with *in vivo* inhalation exposures. Another limitation would be reduced delivery of short-lived reactive compounds to the cells. In the case of aerosols, all constituents might not be delivered to the cell if compounds have poor solubility in the aqueous media. More recently, novel *in vitro* methods have been developed that allow the direct exposure of airborne material to cultured human target cells on permeable porous membranes at the air liquid interface (ALI), with apical surface of the cell exposed directly to smoke/aerosol and the dorsal surface of the cells bathed with cell media (Wieczorek et al., 2023). The effects of HTP and cigarette aerosols on respiratory cells cultured at the ALI can be used to study potential cell injury or activation and the release of bioactive mediators.

With these new testing approaches, new extrapolation methods will be required to interpret and contextualize the *in vitro* assay results. *In silico* simulation is a promising approach to linking *in vitro* inhalation exposure back to *in vivo* exposure (Moreau et al., 2022). Key considerations to enable interpretation and extrapolation of the data to in-life exposure include aerosol particle size distribution, fluid mechanics impacting local deposition rates, and *in vivo* lung morphometries (Tsuda et al., 2013).

The chemical concentration applied *in vitro* that elicits biological activity may be different from the blood or tissue concentration required to elicit a comparable *in vivo* response due to chemical bioavailability, clearance, and other pharmacokinetic (PK) considerations. Quantitative *in vitro* to *in vivo* extrapolation (QIVIVE) can provide an estimate of the likelihood of harmful effects from expected environmental exposures by effectively combining *in silico* and *in vitro* approaches including physiologically based pharmacokinetic (PBPK) modelling, and information on metabolism, transport, binding, and other model parameters from cell- and/or cell derived material-based assays.

PBPK models permit estimation of concentration-time profiles of a compound in various tissues or organs. A whole-body PBPK model contains an explicit representation of the organs that are most relevant to the absorption, distribution, excretion, and metabolism of the test articles due to their physiological/pharmacological function or their volume. The tissues are linked by the arterial and venous blood compartments, and each one of them is characterized by an associated blood-flow rate, volume, tissue-partition coefficient, and permeability. A major advantage of PBPK modeling is the creation of a comprehensive structural representation of the physiology of an organism. The various parameters in the model are either obtained from prior knowledge or calculated from specific and carefully validated formulas (Kuepfer et al., 2016). Inhalation PBPK models coupled to computational dosimetry approaches like the Multiple-Path Particle Dosimetry [MPPD, Applied Research Associates 4300 San Mateo Blvd Albuquerque; (Anjilvel and Asgharian, 1995; Asgharian et al., 1995)] are recommended for development of inhalation exposures and are probably the most effective, practical, and accurate approach (Kolli et al., 2020). The MPPD model predicts the deposition of particles in the entire respiratory tract or in a region of the respiratory tract, in adult and other age groups. These estimates of local dosimetry are usually used to characterize dose-response relationship, extrapolate between species or from *in vitro* assays or predict the distribution of a compound in the body when coupled to PBPK modeling.

In the present study we combined a PBPK model for nicotine with a lung deposition model (MPPD) to better understand the *in vitro* assay results in terms of *in vivo* exposure in humans of nicotine which is used as the biomarker of exposure to cigarette smoke and HTP aerosol.

## 2 Materials and methods

### 2.1 *In vitro* assays

#### 2.1.1 Cell culture

BEAS-2B cells were maintained at 37°C in an atmosphere of 5% CO_2_ in Airway Epithelial Cell Growth Medium (AEGM), that consisted of AEGM (Promocell, C-21060) complemented with SupplementMix (Promocell, C-39165) containing Bovine Pituitary Extract 0.004 mL/mL, epidermal growth factor (10 ng/mL), insulin (5 μg/mL), Hydrocortisone (0.5 μg/mL), Epinephrine (0.5 μg/mL), Triiodo-L-thyronine (6.7 ng/mL); holo-Transferrin (10 μg/mL), Retinoic acid (0.1 ng/mL). Sub-cultivation was performed twice a week in T175 cell culture flasks with a cell seeding density of 8.7E5 and 5E5 cells per flask when cultivated over 3 and 4 days respectively.

#### 2.1.2 Cell seeding and treatment

Cells were seeded on cell culture inserts designed to be used with microscopic analysis technologies (Millicell Cell culture inserts: Millipore PICM01250). In brief: Cells were seeded using 400 µL of a cell suspension with 3.5E5 cells/mL per cell culture insert. The loaded cell culture inserts were placed into 24-well plates filled with 250 µL AEGM medium. Cells were incubated overnight (appr. 18 h) at 37°C and 5% CO_2_ to allow adherence and growth on the cell culture insert membrane. On the next day just before exposure, the medium from the apical compartment of the inserts was removed and the inserts were transferred into new 24-well plates with 250 µL HEPES buffered Dulbecco’s minimal essential medium per well. A 24-well plate with the inserts was placed into one of the exposure chambers of the Smoke Aerosol Exposure *In Vitro* System [SAEIVS, (Wieczorek et al., 2023)]. Smoke/aerosol exposure was executed as described in Table 1. Following exposure, the plate with the inserts was removed from the SAEIVS.

**TABLE 1:** Smoke/aerosolization and sample conditions used with the SAEIVS for the high content screening experiments for the ALI exposure of BEAS-2B cell to derive a MEC.

Test article	Puff interval/volume/profile	Dilution factor 1/x	Number of sticks/devices per run	Runs	Puffs per run	Final puff number applied (^a^)
1R6F	30 s/55 mL/bell shape/vent block	5	3	1	7 (1.4^a^)	7 (1.4^a^)
HTP	30 s/55 mL/bell shape/no vent block	1	3	5	8	40

^a^ The Smoke machine was used according to ISO, 20768.

Cell culture inserts with the treated cells were transferred into new 24-well plates filled with 250 µL AEGM medium. Pre-warmed AEGM medium (400 µL) was added to the cells in the apical compartment of the inserts. Cells were allowed to recover and kept in the incubator for 24 h at 37°C and 5% CO_2_. Medium was removed from both compartments and 200 µL 4% formaldehyde in PBS was added to the apical part of the insert for a 15-min fixation step at room temperature.

To compare the effective concentrations of each product type, a study was designed to assess both the number of puffs delivered to the cells (BEAS-2B) and the amount of nicotine delivered at the cell surface. Separate experiments were adopted using glass plates of the same surface areas as the Millipore inserts (and therefore the cell layer surface) and the same puffing parameters and diluted/undiluted smoke/aerosol from the same product variants. For cell exposure, cigarette smoke (1R6F) was diluted 1:5 (smoke: fresh humidified filtered air) and 1:1 (aerosol: fresh humidified filtered air) for the HTP. The aim of this experiment was to determine the number of puffs required for each product to cause a minimum effective concentration (MEC) for the c-jun cellular marker, measured using high-content screening (HCS) and to determine the number of puffs required to deliver nicotine to the cell surface (using glass plates to collect the deposited material). The marker c-jun was chosen as this was the most sensitive of a panel of selected cellular stress markers used with the HCS technique in-house. The aim was to be as conservative as possible, by using the most sensitive cellular marker using our experience with HCS. The MEC is defined as the lowest effective concentration outside of the background negative control range. The MEC had to exceed the calculated background [=3x median absolute deviation (mad)], that was determined by linear or non-linear regression (decided by the best curve fit). For comparative MEC calculation, the average 3x mad of all treatments for c-jun was used as target value. The Phosphorylation of c-jun (p-c-jun) defines a cellular stress marker which is involved in several signal pathways including proliferation, apoptosis, survival, tumorigenesis, and tissue morphogenesis (Schmeck et al., 2006; Dreij et al., 2010).

#### 2.1.3 Cell surface for nicotine deposition

Previous work has assessed the effect of adding cells (V79 Chinese Hamster lung fibroblast cells and BEAS-2B) to the surface of the glass and compared to the cell-free glass surface. The deposition of nicotine on both surfaces was comparable and so clean glass slides were subsequently used (Wieczorek et al., 2023).

#### 2.1.4 Nicotine evaluation from glass plates and cell medium

Nicotine was quantified using LC-MS/MS method (Internal Standard: Nicotine-d4). Nicotine trapped in cell media and PBS was measured directly without any further sample preparation. Whereas the nicotine trapped on the surface of the glass disc was eluted with isopropanol prior to final measurement.

Nicotine quantification of isopropanol extracts from exposed glass discs and basal medium was conducted using LC-MS/MS (AB Sciex API 6500 QTRAP (SCIEX, Darmstadt, Germany)). For analysis, medium samples were diluted 1:100 with MilliQ water/MeOH (1:1) and 1:1 in the autosampler with the internal standard solution in methanol. A Gemini NX-C18 column (110 Å, 100 × 2.0 mm, 3 µm) (Phenomenex, Aschaffenburg, Germany) was used for the liquid chromatography (oven temperature, 55°C), sample injection volume was 5 µL and the autosampler temperature was 5°C. The eluent gradient was applied according to the following: 0min: 2% B (methanol)/98% A (0.05% acetic acid in water) (flow rate: 400 μL/min); 1.2 min: 65% B/35% A (400 µ/min); 1.5 min: 95% B/5% A (400 µ/min); 2.5 min: 98% B/2% A (400 µ/min); 3.0 min: 98% B/2% A (400 µ/min). The following conditions were used for the mass spectrometry: Ion spray voltage: 4500V, Ion source temperature: 500°C, MRM: 163/132 quantification; 163/106 qualifier. The isopropanol samples were diluted 1:100 with MilliQ water/MeOH (1:1) and without autosampler dilution because internal standards were present in samples extraction and calibration solution. All other measurement parameters were the same as above.

**FIGURE 1:**
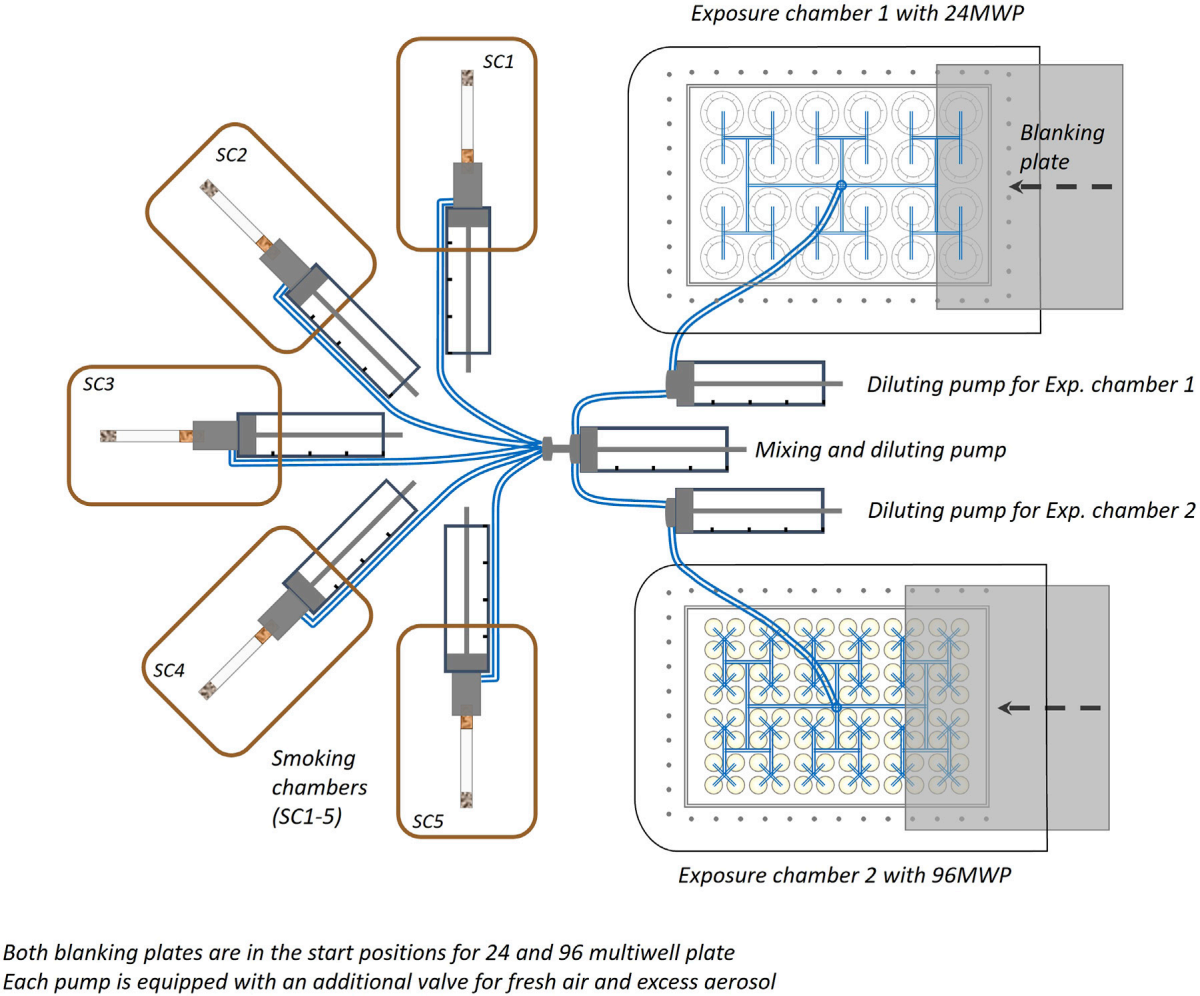
Diagram of the Aerial view of the smoke/aerosol exposure *in vitro* system (SAEIVS). The system consists of five smoking chambers (SCs), which can accommodate cigarettes, electronic nicotine delivery systems and heated tobacco products (in respective runs). The smoke/aerosol generated is then drawn through tubing to a mixing and diluting pump (with charcoal filtered, humidified air). Each exposure chamber also has a further individual diluting pump to allow cells to be exposed different concentrations if required. The exposure chambers can accommodate either 24 or 96 well plates, and smoke/aerosol is delivered to individual wells via ports situated above each individual well. Between the exposure ports and the cell culture plate, a blanking plate can be moved (robotically) to prevent exposure to rows. As the blanking plate moves across the row of wells exposed, preventing subsequent exposure, an increasing dose can be delivered to uncovered rows.

To trigger the p-c-jun response in BEAS-2B cells, the cells were exposed to fresh smoke/aerosol from 1R6F/Pulze used with iD sticks (HTP). For details of the Pulze device and the iD sticks please see Chapman et al. (2023). The BEAS-2B cells were pre-grown on microporous membranes of dedicated cell culture inserts and supplied with medium apically and basally. For exposure to fresh smoke from the Reference Cigarette 1R6F and aerosol from Pulze and iD the cells were switched to ALI conditions (i.e., apical medium was aspirated) where nutrification of the cells is achieved from the lower compartment below the insert-containing well only. The use of the ALI exposure is key. *In vitro* exposure systems that deliver aerosols to the surface of human cells cultured at ALI are of particular importance being the most physiologically relevant exposure route, and highly preferable to using submerged cell lines (Upadhyay and Palmberg, 2018). Upadhyay and Palmberg (2018) identified several factors influencing the successful development of ALI-models including the choice of cell line (preferably human), the source of any primary cells, and the use of co-culture systems consisting of multiple cell types (Clippinger et al., 2016). The use of an ALI system is also considered to be a feasible alternative approach and can be used to implement the “3 Rs principle” replacement, reduction, and refinement of animal usage—in conducting pulmonary toxicity studies (Upadhyay and Palmberg, 2018). The apical surface of the inserts with the cells were exposed directly to fresh aerosol and smoke in the in-house Smoke and Aerosol Exposure *In vitro* System (SAEIVS). This is an in-house built system to enable the delivery of whole aerosols directly to cells at the ALI, being able to deliver different dilutions of aerosol/smoke to two separate cell exposure chambers in under 10 s of generation [for more details and characterization of the system see Figure 1; Wieczorek et al. (2023)]. Following exposure to the aerosol/smoke the cells were covered with recovery medium and were incubated for 24 h before fixation and subsequent antibody staining for p-c-jun.

The study was composed of two parts.

1 - The quantification of phosphorylated c-jun protein in nuclei of BEAS-2B cells after direct exposure to diluted fresh smoke of 1R6F Reference cigarette and to undiluted fresh aerosol of Pulze and iD sticks at the ALI using the SAEIVS. The aim of this part of the study was to determine the p-c-jun MEC for both test articles on a puff basis by means of HCS technology.
2 - A dosimetry approach to determine the amount of nicotine delivered in the SAEIVS per well equipped with inserts corresponding to those used in part 1 of the study. Glass plates were loaded on top of the membranes to mimic the cell surface for smoke/aerosol deposition, with nicotine as the key dosimetry marker. Following exposure of those glass plates, they were extracted with isopropanol and the amount of nicotine trapped per glass plate was measured.

#### 2.1.5 Combination of HCS and nicotine data

Following the determination of MEC and finalization of nicotine dosimetry, the data were combined to determine the MEC on a nicotine basis. To this end the MEC_puff_ from the HCS approach was used to determine the MEC_nic_ by using the MEC_puff_ in the equation of the nicotine dosimetry.

### 2.2 MPPD modeling

Respiratory tract deposition models consider the anatomic structure of the respiratory tract, the air flow patterns and the aerodynamic characteristics of the particles to predict the deposited dose in each region. Lung deposition was calculated using the MPPD model (MPPD version 3.04) available from the Applied Research Associates webpage (https://www.ara.com/mppd/).

The MPPD model includes both human and rat respiratory tract models of the deposition and clearance of spherical particles. It predicts the deposition and clearance of monodisperse and polydisperse aerosols in the respiratory tracts for particles ranging in size from ultrafine (1 nm) to coarse (100 µm). Several factors can influence deposition including the concentration of the chemical/particles in the air, the aerodynamic characteristics of particles, the frequency and duration of exposure, the physiological inhalation parameters such as the anatomical structure of the respiratory tract, breathing patterns, and interaction with other airborne particles (Rozman and Klaassen, 2001).

In the simulations presented here, the stochastic model was parameterized to have upright body positioning and oral breathing at constant exposure conditions. Different scenarios of exposure were tested and deep breathing at resting, with a short breath hold chosen for the simulations. This corresponds to a breathing rate of 12 breaths per min, a tidal volume of 1.3 L, an inspiration fraction of 0.5, a pause fraction of 0.1 and finally inhalation and exhalation times of 2.5 and 2 s, respectively. This scenario was chosen as the best one based on smoking habits (McEwan et al., 2019). In his review, Bernstein (2004) describes a specific pattern of smoking with 2 phases: The initial puff is first taken into the mouth and after a pause of 1–4 s, the smoke is then inhaled into the lungs.

We used the stochastic lung model as it represents asymmetric structures of the tracheobronchial region of a human lung. This model describes the randomness and asymmetry of the airway branching system. This lung model is based on distributions of morphometric parameters such as length, diameter, branching angle, cross-sectional area of the daughter tubes, gravity angle, and correlations between these parameters as a function of airway generation (Asgharian et al., 2001). This provides more realistic lung geometry than the symmetric lung models in MPPD (Yeh-Schum Single path, Yeh-Schum 5-Lobes, PNNL symmetric model or Weibel symmetric model). There are 10 models within the stochastic lung model ordered in size (total number of airways) from the smallest to the largest and the approximate size percentile of each lung. The 60th percentile human stochastic lung model was used for our simulation.

Concerning the particle size diameter for IVIVE, the mass median aerodynamic diameter (MMAD) was set to 0.8 µm with a geometric standard deviation (GSD) of 1.3 for 1R6F and 0.7 µm with a GSD of 1.5 for HTP based on Schaller et al. (2016). GSD represents the geometric standard deviation, and the larger the GSD value, the greater the spread of the aerosol diameter of the particles.

We also chose to incorporate an inhalability adjustment to the simulations. This is an adjustment for particle size larger than 8 µm. The probability that these are inhaled is less than 1 and decreases with increasing particle size. This correction is used to account for expected inertial deposition of the larger particles (Asgharian et al., 1995).

### 2.3 PBPK modeling

#### 2.3.1 Model development and structure

The absorption, distribution, metabolism, and excretion (ADME) properties of nicotine have been previously studied in different species (Benowitz et al., 2009a; Benowitz et al., 2009b). Nicotine is metabolized quickly in the liver, primarily by cytochrome P450s (CYP2B6 and CYP2E1) and has relatively low plasma protein binding (Benowitz et al., 2009a; Benowitz et al., 2009b). Large venous blood nicotine concentrations are produced within minutes after nicotine inhalation as the lungs offer a large surface area for absorption and a favorable dissolution pH of 7.4. Several PBPK models have been developed for nicotine (Plowchalk et al., 1992; Robinson et al., 1992; Teeguarden et al., 2013; Haghnegahdar et al., 2018).

**FIGURE 2:**
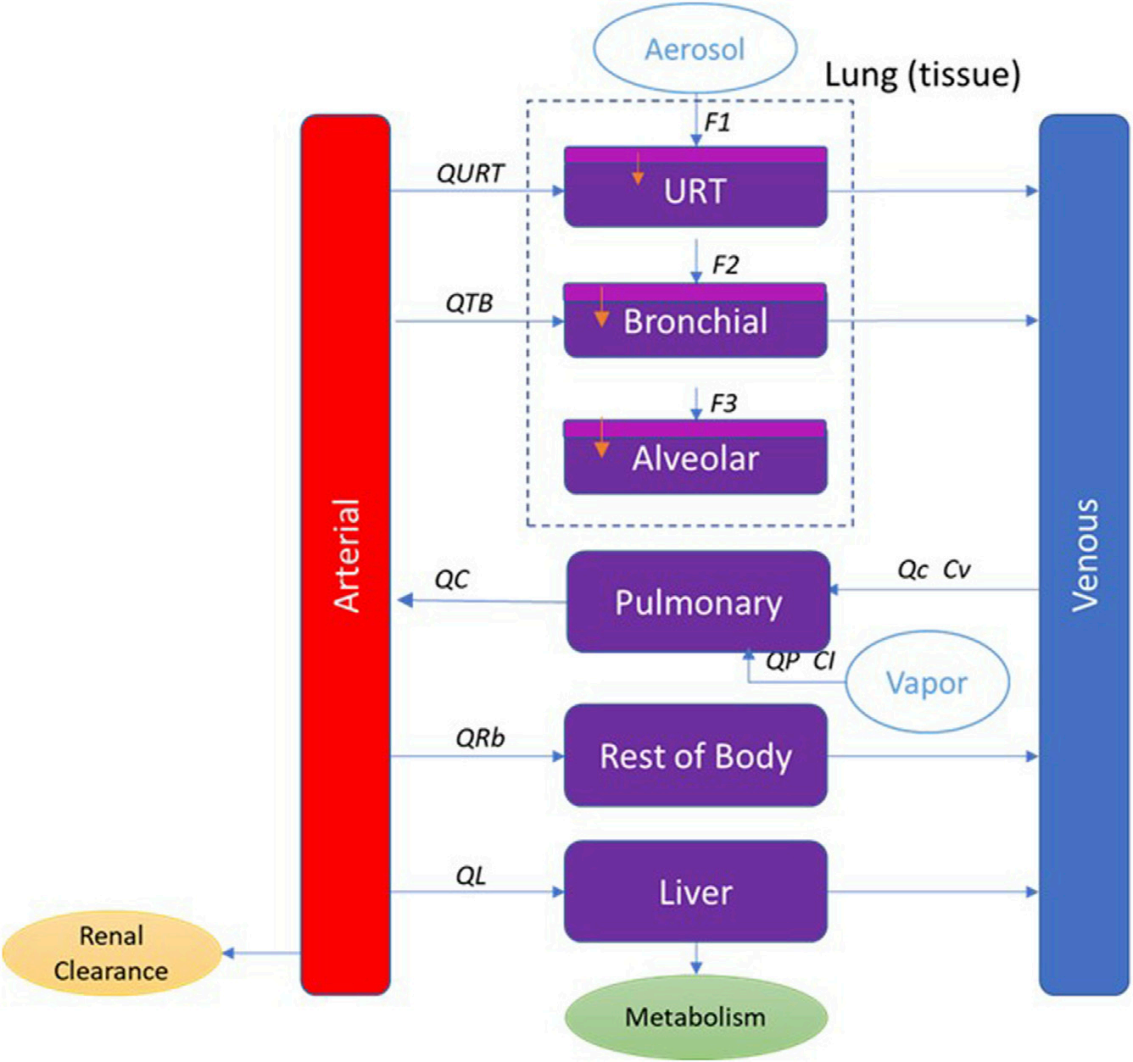
PBPK model schematic for nicotine showing the representation of the main organs considered with various sub-compartments in the lung for inhalation exposure. QL, QRb, QC, QTB, and QURT refer to blood flow to each tissue compartment. QP refers to the alveolar ventilation rate. Cv refers to the venous concentration and CI to the inhaled vapor concentration. F1, F2, and F3 refer to the fraction deposited in each region of the respiratory tract. In the respiratory tract (URT, bronchial and alveolar), the light purple represents the mucus and epithelium, and the dark purple represents the submucosa region where blood perfusion occurs.

The PBPK model developed here has perfusion-limited compartments for liver and lung and lumped compartment for the remaining tissues (rest of the body). Inhalation of aerosols is a complex process, and PBPK models with regional lung compartments are more descriptive. Here, we use a multicompartment respiratory tract model. Based on anatomical location and function, the respiratory tract is divided into three regions: upper respiratory tract (URT), trachea-bronchial region (TB) and alveolar region. There is also a pulmonary compartment which represents the gas exchange region of the lung. To include processes that transport the absorbed nicotine across the anterior respiratory tract compartments, these regions are further divided into a two-layered substructure: an epithelial cell layer with mucus, and a submucosal tissue layer. The submucosal tissue layer has blood perfusion and clears the absorbed nicotine from the airway compartments. Consistent with common practice, the tissue compartments are well-mixed reservoirs. Exposure is characterized in each lung (inhalation) compartment based on calculated deposition rates. Elimination occurred from both the liver and the blood compartment and was represented by hepatic and renal clearance, respectively. The model structure is shown schematically in Figure 2.

The physiologically-based biokinetic (PBBK) model was developed in Berkeley Madonna software (version 10.1.3; University of California, Berkeley, CA; www.berkeleymadonna.com). Model equations are in Supplemental 1.

#### 2.3.2 Model parameters

Parameters used in the current model are summarized in Table 2. Tissue blood flows are from our ScitoVation database for an adult male (internal database not shown), except for the blood flow to the upper respiratory tract (URT) that was set according to Campbell et al. (2015) and the blood flow to the alveolar region that was set according to Butler (1992). The ScitoVation database includes age-specific physiological parameters for body weight (BW), cardiac output, tissue weights (volumes), and tissue blood flows, and are adapted from published life-stage models (Wu et al., 2015; Song et al., 2016; Ruark et al., 2017).

**TABLE 2:** Physiological and biochemical parameters used for the nicotine PBPK model.

Parameters	Parameters description	Values		References
		Original	Fitted	
BW	Body weight—kg	81.2	—	Scitovation database
QC	Cardiac output—L/h	423.37	—	Scitovation database
QLc	Blood flow to the liver—Fraction of QC	0.235	—	Scitovation database
QURTc	Blood flow to the URT—Fraction of QC	0.00247	—	Campbell et al. (2015)
QTBc	Blood flow to the TB region—Fraction of QC	0.0075	—	Campbell et al. (2015)
QALVc	Blood flow to the pulmonary region—Fraction of QC	0.0067	—	Butler (1992)
VLc	Liver volume—Fraction of BW	0.0197	—	Scitovation database
VArtc	Arterial blood volume—Fraction of BW	0.0142	—	Scitovation database
VVc	Venous blood volume–Fraction of BW	0.0427	—	Scitovation database
SAurt	URT surface area—cm^2^	154.8	—	MPPD
SAtb	TB surface area—cm^2^	4440	—	MPPD
SAta	Transitional airway surface area—cm^2^	6220	—	Sarangapani et al. (2002a)
SApulm	Pulmonary region surface area—cm^2^	700146.6	—	MPPD
TmucepithURT	Mucus and epithelium thickness in the upper respiratory tract—cm	0.006	—	Sarangapani et al. (2002a)
TmucepithTB	Mucus and epithelium thickness in the tracheo-bronchial region—cm	0.0066	—	Sarangapani et al. (2002a)
TmucepithTA	Mucus and epithelium thickness in the transitional airway—cm	0.001	—	Sarangapani et al. (2002a)
TmucepithPULM	Mucus and epithelium thickness in the pulmonary region—cm	0.0005	—	Sarangapani et al. (2002a)
TURT	Submucosa thickness—cm	0.012	—	Bogdanffy, personal communication
TTB	Submucosa thickness—cm	0.0132	—	Bogdanffy, personal communication
TTA	Submucosa thickness—cm	0.002	—	Bogdanffy, personal communication
TV	Tidal volume—L	1.3	—	Brown et al. (1997)
DS	Dead space in the lung—L	0.15	—	Brown et al. (1997)
BR	Breathing rate—/h	720	—	Brown et al. (1997)
DLc	Nicotine diffusion—cm^2^/h	4.87E-9	—	QSAR
PL	Liver: blood partition coefficient	7.8	7	Teeguarden et al. (2013)
PURT	URT: blood partition coefficient	2	1.23	Robinson et al. (1992)
PTB	TB: blood partition coefficient	2	0.1	Robinson et al. (1992)
PALV	ALV: blood partition coefficient	2	0.1	Robinson et al. (1992)
PRB	Rest of the body: blood PC	7.8	1.48	Teeguarden et al. (2013)
Fu	Plasma unbound fraction	0.95	—	Robinson et al. (1992)
CLr	Renal clearance—L/h	4.25	—	Yamazaki et al. (2010)
CL_int_	Intrinsic clearance—L/h	63	128.5	Robinson et al. (1992)
FURT	Particle fraction deposited in the URT	0.014	—	MPPD
FTB	Particle fraction deposited in the TB	0.07	—	MPPD
FPULM	Particle fraction deposited in PULM	0.1629	—	MPPD
Fvapor	Vapor fraction for Cigarette	0.0247	0.20	Haghnegahdar et al. (2018)
BPR	Blood plasma ratio	1	—	Values range from 0.82 to 1.2 so estimation at 1

Volumes were set to values from the ScitoVation database except for the lung volume. Tissue volumes for the three respiratory-tract compartments were estimated by multiplying the appropriate surface area with the tissue thickness. The surface area of the three lung regions was estimated using a standard lung model (MPPD) that quantifies airway length and diameter on a generation-by-generation basis. Epithelial thickness was obtained from Sarangapani et al. (2002b). The submucosal thickness was assumed to be approximately twice the epithelium thickness, based on histological sectioning (Matthew Bogdanffy, personal communication).

Chemical-specific parameters such as hepatic intrinsic clearance and renal clearance were obtained from previously published PBPK model from Robinson et al. (1992) and Yamazaki et al. (2010). Robinson et al. (1992) estimated a hepatic clearance of 1.09 L/min, using a PBPK model calibrated with *in vivo* human data.

Tissue: blood partition coefficients (PC) are defined as the ratio of the concentration of a test chemical in two media (i.e., tissue and plasma), once equilibrium is reached. PCs are important determinants of the disposition of chemicals in different tissues. Liver PC was from Teeguarden et al. (2013). The other PCs (the rest of the body and lung compartments) were fitted to the *in vivo* human data. Plasma protein binding of approximately 5% has been reported by Robinson et al. (1992).

Nicotine permeability was taken from literature. There was some variability in reported values: 1.0E-4 cm/s from Gowadia and Dunn-Rankin (2010), 1.28E-4 cm/s from Waddell and Marlowe (1976), 2.5E-5 cm/s and 1.14E-5 cm/s from a QSAR model used by Symcyp. We opted to use the QSAR value as it was giving a better fit with the *in vivo* PK data. The nicotine permeability was multiplied by the tissue thickness in the experiment to derive a diffusion coefficient in cm^2^/s.

The fraction of particles deposited in each of the lung compartments was estimated using the MPPD model. To validate the model, we simulated individuals with deep breathing at rest with a short breath hold. Most of the studies used to validate the model did not provide direct information about the scenario of exposure. In most of the studies, volunteers were at rest, i.e., reading quietly, working, or engaging with social media. Based on smoking behavior found in different publications (Pichelstorfer et al., 2016; McEwan et al., 2019), a deep breath at resting and a short breath hold seemed to be the more appropriate scenario to reflect the human *in vivo* data used to validate the model.

The fraction of vapor inhaled for cigarettes was fitted to the data. Here the particle size diameter, the count median diameter (CMD) was set to 163 nm with a geometric standard deviation (GSD) of 1.44 for cigarettes based on Fuoco et al. (2014) and Sosnowski and Kramek-Romanowska (2016). These values were used for the calibration and validation of the model.

#### 2.3.3 Model calibration and validation

The performance of the model was first evaluated using the *in vivo* pharmacokinetic (PK) data (plasma concentrations between 0–4 h post exposure) from McEwan et al. (2019) where 48 healthy subjects smoked a single assigned test cigarette and had blood drawn on the morning of each visit. In the afternoon, the smoking behavior assessment was carried out with a single use of the same test product.

The model was also validated using 2 other *in vivo* PK datasets from Picavet et al. (2016), and Digard et al. (2013).

Picavet et al. (2016) assessed the PK of nicotine after a single use of cigarette in 28 healthy smokers. The cigarettes assessed in this study were non-menthol, manufactured, commercially available cigarettes, with a maximum ISO yield of 1 mg nicotine per cigarette. The pharmacokinetics of nicotine were measured on the days of single use. The first blood sample was collected within 15 min before a single use of the allocated product in the morning, and then at 2, 4, 6, 8, 10, 15, 30, 45, and 60 min, and at 3, 4, 6, 9, 12 and 24 h.

Digard et al. (2013) conducted a study with 20 healthy cigarette users. A cigarette was smoked, and blood samples taken at intervals over 120 min. The subjects were instructed to smoke the cigarette naturally according to their usual smoking behavior for 5 min or until the cigarette had been smoked to 30 mm from the mouth end (if this occurred in less than 5 min), at which point the cigarette was extinguished.

All the puffing scenario and dose (1 mg nicotine) used in each of these studies were the same with a puff duration of 2.3 s, puff interval of 30 s, puff volume of 69.5 mL and number of puffs per cigarettes of 14. Puff duration, number of puffs and puff volume for cigarette were based on McEwan et al. (2019). The puff interval was assumed to be 30 s (Bernstein, 2004).

The dose of nicotine in one cigarette was assumed to be 1 mg (the amount varies between brands but is usually between 0.7 and 1 mg) (EU, 2014).

#### 2.3.4 Normalized sensitivity analysis

To evaluate the relative impact of each of the model parameters on nicotine plasma maximal concentration (C_max_) and area under the curve (AUC), a sensitivity analysis was performed. The sensitivity coefficient (SC) was calculated according to the equation below (Teeguarden et al., 2005).

SC=Fractional change in model output/Fractional change in parameter

Each parameter was individually increased by 1% of its original value, with the other parameters held constant. The larger the absolute value of the sensitivity coefficient, the more important the parameter. A normalized sensitivity coefficient of 1 represents a 1:1 relationship between the change in the parameter and the internal dose metric of choice. A negative SC indicates the given parameter influences the dose metric in an inverse direction. The SCs are grouped into one of three categories: namely, high (absolute SC values greater than or equal to 0.5), medium (absolute SC values greater than or equal to 0.2 but less than 0.5), or low (absolute SC values greater than or equal to 0.1 but less than 0.2), according to the IPCS guideline (WHO, 2010).

### 2.4 *In vitro* to *in vivo* extrapolation

**FIGURE 3:**
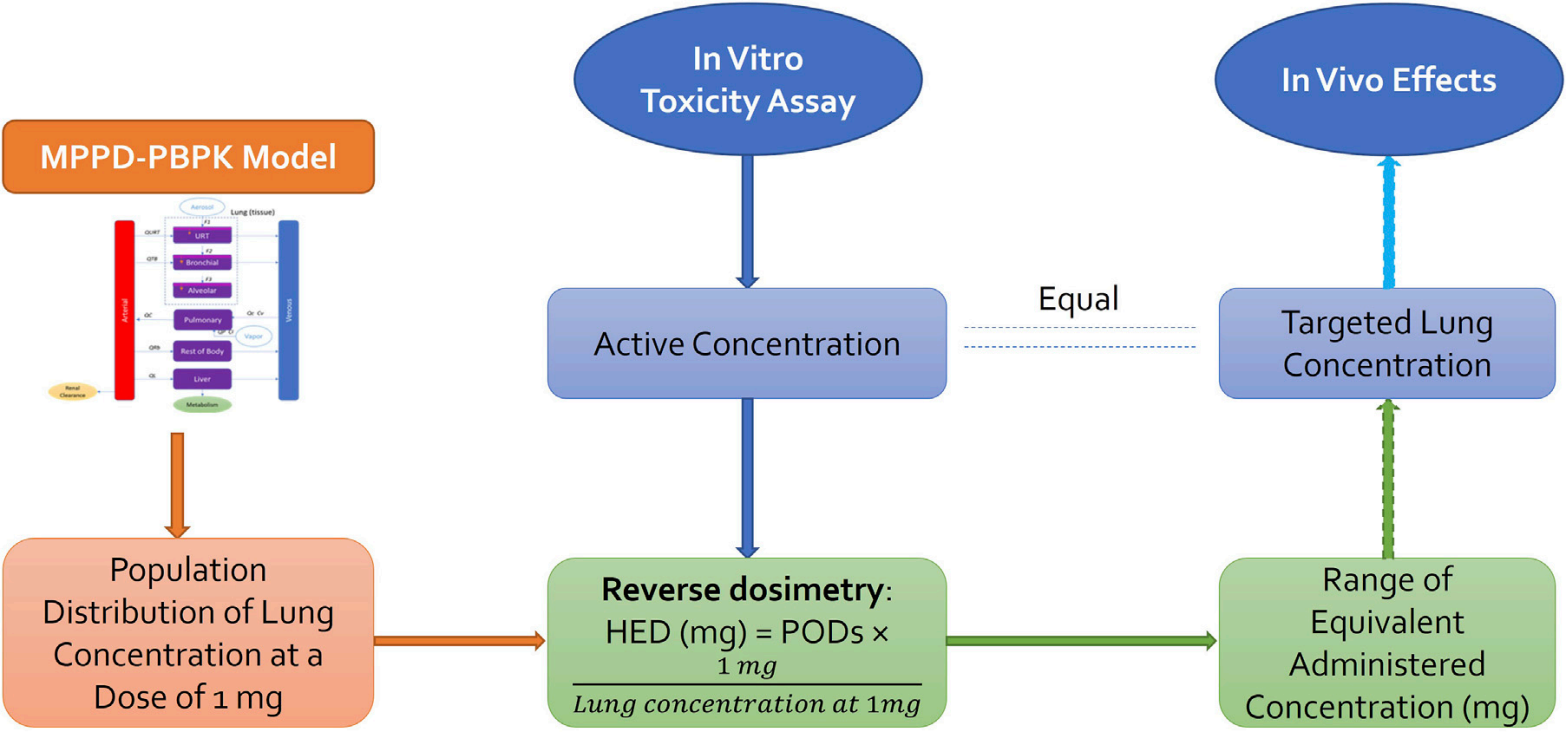
*In vitro* to *in vivo* extrapolation. Physiological parameters as well as parameters describing ADME processes of the chemical through the system were used to develop a PBPK model that can be used to predict the population distribution of lung concentration from any given daily dose. Reverse dosimetry predicts administered doses equivalent to *in vitro* active concentration, which can be compared to the *in vivo* measurements [Adapted from Bell et al. (2018)].

The goal was to estimate the Human Equivalent Concentration (HEC) from *in vitro* assays and nicotine plasma concentration. Three steps were followed to derive a tracheobronchial epithelium concentration equivalent to an MEC from an *in vitro* assay (Figure 3).

- Estimate the fraction deposited for a scenario type with MPPD.
- Use the fraction deposited from MPPD in the PBPK model and by reverse dosimetry derive the exposure concentration that matches the estimated *in vitro* deposition POD. BEAS2B cells were used so we used the amount deposited in the tracheobronchial region to perform reverse dosimetry (AT2 in the model code).
- Simulate plasma concentration using the PBPK model.

## 3 Results

### 3.1 *In vitro* assays

**FIGURE 4:**
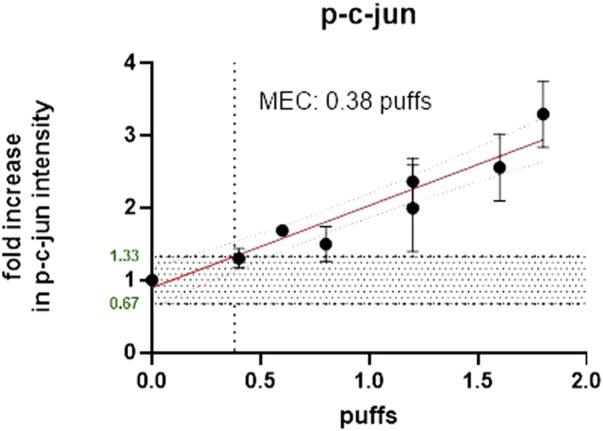
Number of puffs of 1R6F to reach the MEC for p-c-jun using high content screening.

**FIGURE 5:**
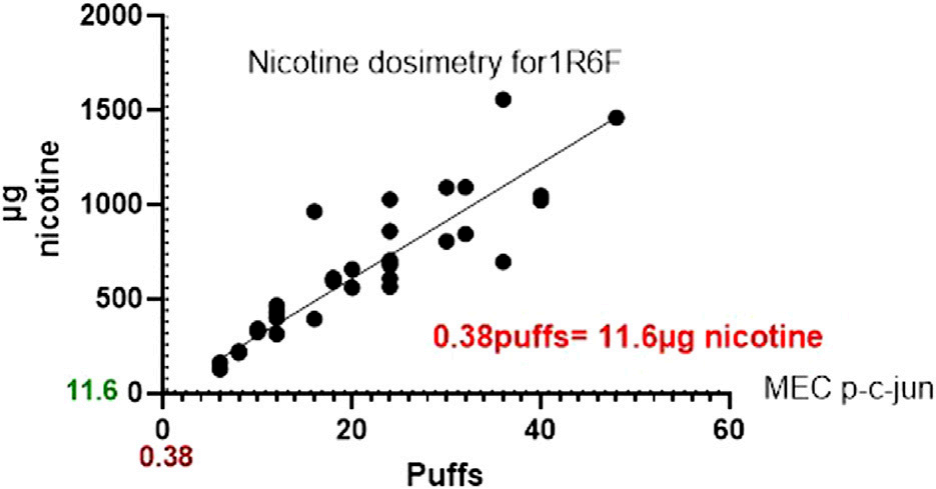
Graph comparing the nicotine mass deposited by 1R6F on to glass slides at the corresponding number of puffs required to the reach the MEC using High Content Screening.

1R6F exposure of BEAS2B to air-diluted smoke (1:5) with a subsequent recovery of 24 h was performed. Figure 4 shows the calculation of the minimum effective concentration (MEC), and Figure 5 is the calculation of nicotine mass corresponding to the MEC threshold captured on glass plates.

**FIGURE 6:**
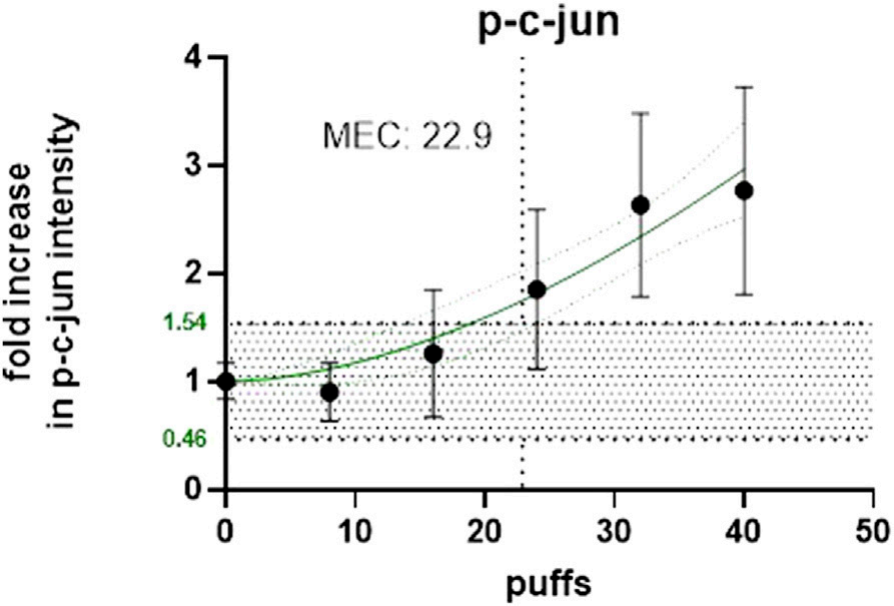
Number of puffs of HTP required to reach the MEC for p-c-jun using High Content Screening.

**FIGURE 7:**
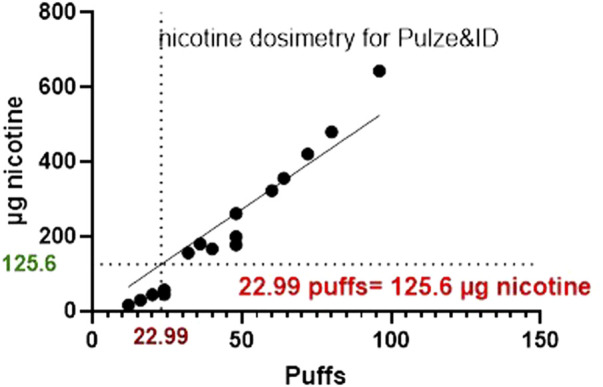
A graph comparing the nicotine mass deposited by HTP on to glass slides at the corresponding number of puffs required to the reach the MEC using HCS.

The same experiments were also conducted for HTP and Figures 6, 7 below shows the p-c-jun HTP results, with the MEC and number of puffs required to reach the MEC.

### 3.2 MPPD modeling

Cigarette deposition fractions in the respiratory tract used to calibrate and validate the PBPK model can be found in Table 2. With a CMD of 0.163 µm and GSD of 1.44 and according to the scenario of exposure described in the method section, the deposition fractions in the head, the trachea-bronchial region and the pulmonary region were 0.0147, 0.0673 and 0.1629, respectively. The total deposition fraction equaled 0.2449.

### 3.3 PBPK modeling

#### 3.3.1 Model calibration and evaluation

**FIGURE 8:**
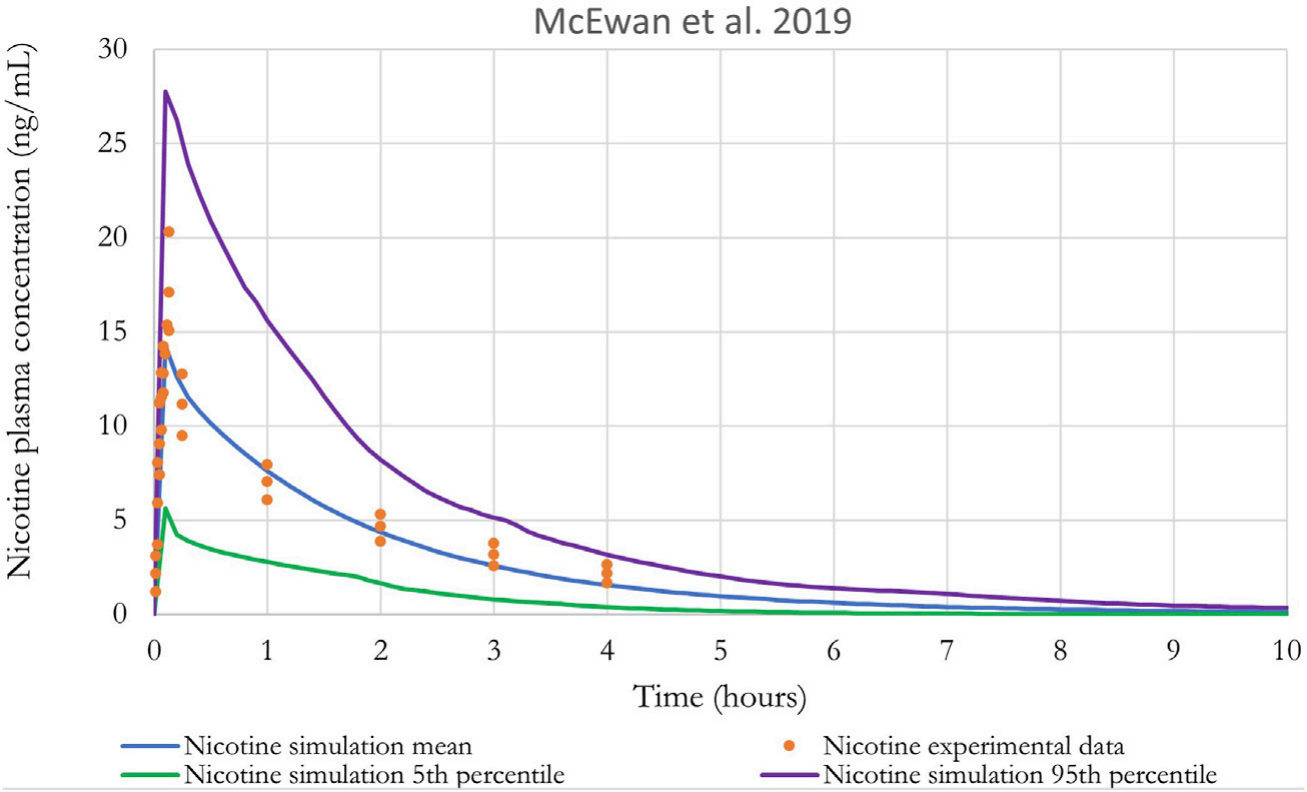
Nicotine plasma concentration in adult humans following inhalation exposure (1 mg). The solid line is the simulated venous concentration in ng/mL and the red circles are the PK data from McEwan et al. (2019) in ng/mL (mean ± SEM).

Figure 8 shows the results of the performance of the model when evaluated using the *in vivo* PK data for nicotine from McEwan et al. (2019). A qualitative evaluation of the agreement between experimental plasma concentration and simulations was conducted through visual inspection of the time-course, where good agreement is generally defined as simulations falling within a factor of two of the data (EPA, 2006). In addition, IPCS (2010) guidance on “Principles of Characterizing and Applying PBPK Models in Risk Assessment” states that “In PBPK modeling, predictions that are, on average, within a factor of 2 of the experimental data have frequently been considered adequate”.

**FIGURE 9:**
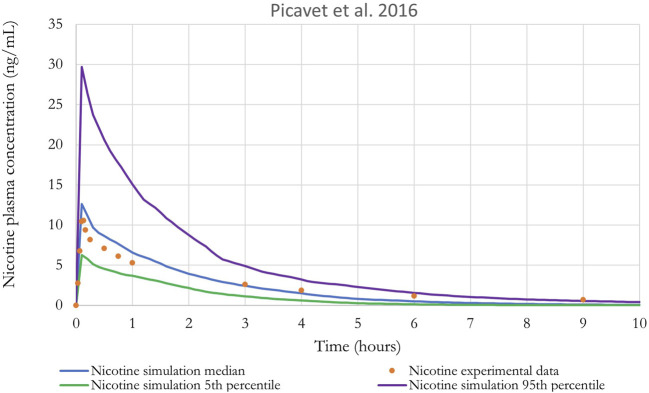
Nicotine plasma concentration in adult humans following inhalation exposure (1 mg). The solid line is the simulated venous concentration in ng/mL and the red circles are the PK data from Picavet et al. (2016) in ng/mL.

**FIGURE 10:**
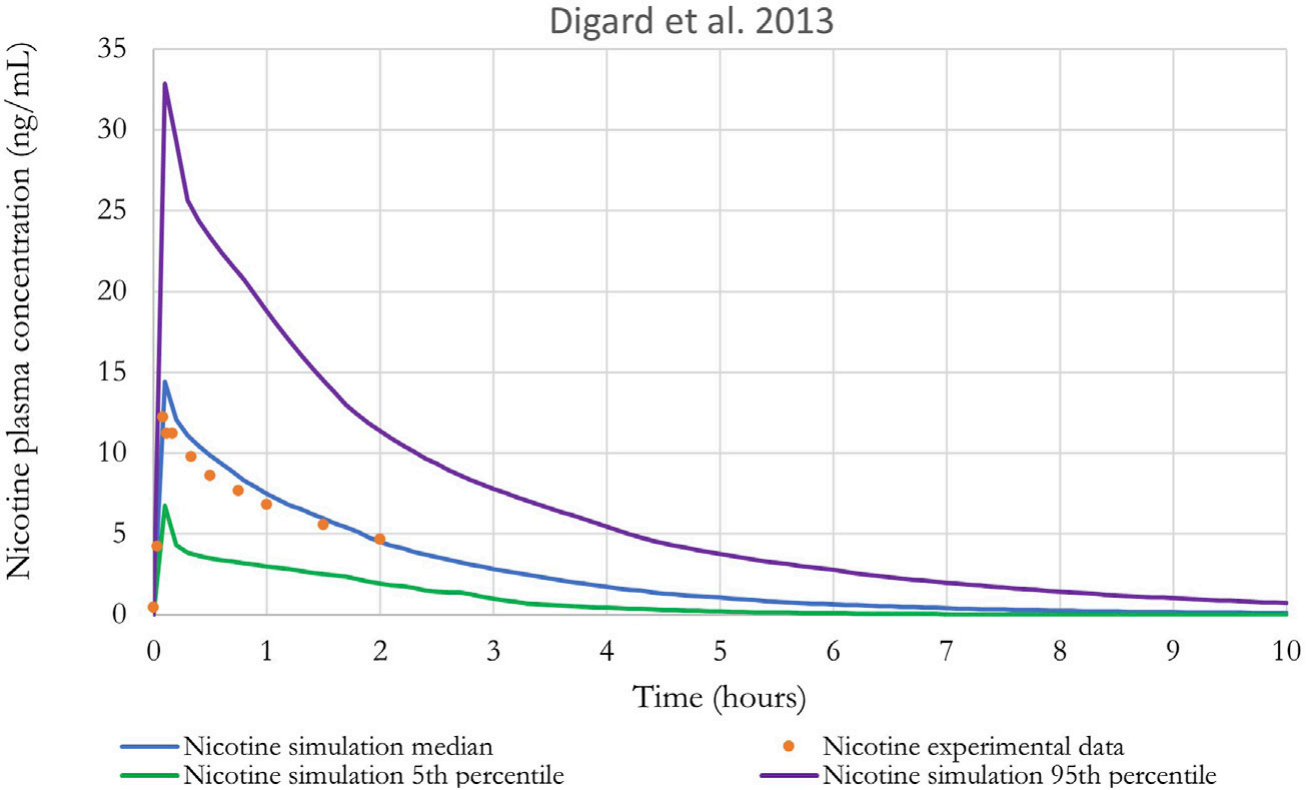
Nicotine plasma concentration in adult humans following inhalation exposure (1 mg). The solid line is the simulated venous concentration in ng/mL and the green circles are the PK data from Digard et al. (2013) in ng/mL.

Monte Carlo (MC) analysis was also conducted to investigate the population variability. Only the sensitive parameters from the sensitivity analysis were varied. Partition coefficients, body weight, breathing rates, and metabolic constants were simulated as log normally distributed; and cardiac output, blood flows, tissue volumes and parameters related to puffing scenario (puff volume, puff duration, puff interval) were simulated as normally distributed. The coefficients of variation (CV) for partition coefficients were 30%, a CV of 22% and 16% were used for the body weight and for the blood flow to the liver, respectively (Price et al., 2003), while a CV of 30% was assumed for the remaining model parameters. The distributions were truncated at 2 Standard deviations (Clewell and Clewell, 2008) to ensure physiological plausibility. Parameters distribution can be found in Supplementary Table S1. Monte-Carlo simulations were performed with 100 iterations to perform population-level simulations. Mean or median plasma concentration as well as the 5th and 95th percentiles are graphically represented in Figures 8–10. For IVIVE, Monte-Carlo simulations were performed with 1,000 iterations to perform population-level simulations.

The performance of the model was also evaluated using *in vivo* nicotine PK studies with cigarettes (Digard et al., 2013; Picavet et al., 2016). Simulations results can be found in Figures 9, 10. The PBPK model predictions were consistent with the nicotine concentrations in plasma in adult humans.

#### 3.3.2 Sensitivity analysis

The sensitivity of model parameters was calculated with the human PBPK model for the plasma maximum concentration (C_max_) and area under the curve (AUC) after low and high inhalation doses of nicotine (1.2 and 12 mg). Results from the normalized sensitivity analysis are in Supplementary Figure S1. The most sensitive parameters are the ones related to inhaled dose: puff duration, puff interval, breathing rate, tidal volume, puff volume and fraction of vapor. The analysis showed that physiological parameters, including body weight, cardiac output and blood flow to the liver are medium to highly sensitive. The parameter for particle deposition in the pulmonary region is also a medium-sensitivity parameter. Note that hepatic metabolism parameters showed no sensitivity for plasma C_max_ but medium sensitivity for the plasma AUC. This does not mean that metabolism is not influential in plasma C_max_, but indicates that metabolism is so efficient, i.e., CL_int__*in vivo* very much exceeds liver plasma flow and a 1% increase in the parameter value would not make a difference, as it is largely limited by liver plasma flow, which is one of the most sensitive parameters.

The uncertainty of a model reflects the level of confidence in model predictions. The structure of the model is based on previously published models (Plowchalk et al., 1992; Robinson et al., 1992) where we included a more complex description of the lung to be able to link a dosimetry model (MPPD). The vapor fraction for cigarette was fitted to the data. For vapor absorption, we assumed that vapor would be completely absorbed in the deep lung. A more complex description of vapor absorption along the respiratory tract may provide simulations more similar to the observed data.

### 3.4 *In vitro* to *in vivo* extrapolation

The mass median aerodynamic diameter (MMAD) was set to 0.8 µm with a geometric standard deviation (GSD) of 1.3 for 1R6F and 0.7 µm with a GSD of 1.5 for HTP based on Schaller et al. (2016). For the PBPK model, we used values from McEwan et al. (2019) and O'Connell et al. (2019) with a puff duration of 2.3 s, a puff interval of 30 s, a puff volume of 69.5 mL and a number of puffs per cigarettes of 10. Results from MPPD modeling are presented in table 3.

**TABLE 3:** Nicotine fraction deposited for 1R6F and HTP in MPPD.

Deposition	Head	Tracheobronchial region	Pulmonary region
1R6F	0.0082	0.0608	0.1454
HTP	0.0089	0.0615	0.1456

We used the PBPK model and predicted the dose of nicotine per cigarette necessary to reach the MEC for both 1R6F and HTP. Monte Carlo simulations were used to estimate median, 5th and 95th percentiles for the amount of nicotine in the epithelium of the tracheobronchial region (AT2) for individuals exposed to the same, fixed dose (1 mg). Since the upper 95th percentile individuals have higher amounts for the same exposure, they are an example of a sensitive population. Then, reverse dosimetry predicted administered doses equivalent to the active concentrations from the *in vitro* assays (MEC of 0.072 and 0.77 µmol for 1R6F and HTP, respectively). The exposure concentration necessary to reach the MEC are in Table 4, and equivalent plasma concentrations are shown in Table 5.

**TABLE 4:** Human equivalent exposure concentrations predictions using MPPD-PBPK modeling and the MEC values reported in Figures 4–6 and typical cigarettes and HTP nicotine content for comparison.

	MEC (µmol)	HEC (mg nicotine/stick) median (5th-95th percentiles)	Nicotine concentration (mg nicotine/stick)	Margin of exposure (MOE)
1R6F	0.072	0.288 (0.25–0.337)^a^	1.85	0.16
HTP	0.77	3.05 (2.64–3.58)^a^	1.03	2.96

^a^ Monte-Carlo simulations were performed with 1,000 iterations as described in model evaluation.

**TABLE 5:** Plasma concentrations of nicotine are predicted using MPPD-PBPK modeling, and the HEC values reported in Table 4 and blood concentrations predicted after exposure to the classic nicotine content.

	HEC (mg nicotine/cig)	Plasma concentration after 1 stick (ng/mL)^a^	HEC at steady state (mg nicotine/10 sticks)	Plasma concentration at steady state (ng/mL)^a^
1R6F	0.288	4.16 (1.84–10.02)	2.88	9.38 (4.34–21.42)
1R6F	1.85^b^	28.18 (11.85–65.74)	18.5	60.83 (27.17–139.80)
HTP	3.05	46.48 (20.09–110.98)	30.5	101.43 (46.01–228.11)
HTP	1.03^b^	15.50 (6.47–38.79)	10.3	34.30 (15.32–77.45)

^a^ Median (fifth-95th percentile).

^b^ Nicotine concentration in the Smoke/Aerosol from cigarettes or HTP sticks.

To derive the plasma concentration, we used two scenarios, one where only one cigarette is consumed (at the HEC for both cigarettes and HTP stick and at 1.03 mg nicotine for comparison with nicotine content in cigarettes or 1.85 mg nicotine for HTP stick) and a second scenario where 10 cigarettes are consumed over time (at the HEC × 10 for the total dose of nicotine over the 10 sessions and at 10.3 mg or 18.5 mg nicotine which means 1.03 mg nicotine per cigarette or 1.85 mg nicotine per HTP stick × 10 sessions). For each session, the parameters used were that a cigarette was smoked entirely after 10 puffs, a single puff every 30 s (5 min) and a single cigarette was smoked every hour.

Table 4 shows the margin of exposure (MOE), which is the ratio between the nicotine content necessary to reach the MEC and the classic nicotine content of each cigarette. Results mean that after smoking the equivalent of 1/6th of a 1R6F cigarette, the MEC is already reached. However, for HTP, puffing almost 3 HTP sticks at the same time would be required to reach the MEC.

Plasma concentrations predicted after exposure to the classic nicotine content (1.85 and 1.03 for 1R6F and HTP, respectively) and after exposure to the nicotine content at the MEC are in Table 5. Concentrations in plasma are reported after exposure to a single cigarette/stick and after 10 cigarettes/sticks to approximate a plasma steady state for nicotine. Table 5 shows that nicotine plasma concentrations range from 4 to 28 ng/mL after exposure to 1 stick of 1R6F at the MEC and at a nicotine concentration of a classic cigarette, respectively. For HTP, nicotine plasma concentrations range from 15.5 to 46.5 ng/mL after exposure to 1 stick at a nicotine concentration of a classic HTP and at the MEC, respectively.

When a steady state is reached after 10 cigarettes or 10 HTP sticks (1R6F and HTP), simulations suggest that plasma concentrations have doubled. The plasma concentration of nicotine is not higher because between each stick, nicotine is rapidly eliminated.

## 4 Discussion

To better understand the applicability of *in vitro* assay results in terms of *in vivo* exposure in humans, it is necessary to develop computational tools to describe the PK of nicotine, a key biomarker of exposure to cigarettes and HTPs. In this study, we have combined a PBPK model for nicotine with a lung deposition model (MPPD) to better predict nicotine deposition and to model the pharmacokinetics of nicotine with use of either a HTP or cigarette. We have parameterized and exercised the MPPD model to characterize particle deposition in the respiratory tract and developed a PBPK model for nicotine that was validated by comparisons with human data from *in vivo* clinical studies. Finally, we estimated Human Equivalent Concentration (HEC) and plasma concentrations based on minimum effective concentration (MEC). This was derived by exposing BEAS-2B cells to 1R6F or HTP diluted smoke/aerosol and detecting c-jun activation using high content screening to then calculate the MEC as the point of departure.

Regarding the health consequences associated with tobacco use, several biomarkers of exposure for smoking have been reported (Benowitz et al., 2009b; Chang et al., 2017). In this study, we chose to measure nicotine as it has been studied extensively and is routinely measured in human clinical studies investigating exposure to nicotine-containing products and because of its relative stability for measurement (Crooks et al., 2013). Overall, the MPPD-PBPK model adequately recapitulated the *in vivo* kinetic data of nicotine from human studies with cigarettes. Good agreement with model predictions, generally within a factor of two of the data, was obtained. IPCS (2010) guidance on “Principles of Characterizing and Applying PBPK Models in Risk Assessment” states that “In PBPK modelling, predictions that are, on average, within a factor of 2 of the experimental data have frequently been considered adequate”.

One advantage of PBPK models is their potential to account for population variability. Interindividual variations in metabolism is usually well-documented in humans (Tyndale and Sellers, 2002; Hukkanen et al., 2005). Other causes of variability include the absorption of nicotine (different scenario of exposure depending on product uses) or differences in products used for nicotine delivery (e.g., cigarettes vs. HTP) and the physico-chemical characteristics of particles. The impact of changes in particle size through MPPD modelling or in scenarios of exposure (smoking habits) can be studied with a PBPK model. Supplementary Figure S2 in supplemental, for example, shows the regional deposition fractions of inhaled aerosol particles as a function of different breathing scenarios (Supplementary Material S1, Supplementary Table S2). MPPD calculations of deposition in the head and tracheobronchial regions were very similar for many breathing patterns. However, deposition in the pulmonary region is highly dependent on breathing pattern and increases with longer breath hold and deeper, slower breath. In all cases, the total deposition is less than 40%. Concerning smoking habits, Jones et al. (2020) reported that the puffing data also provides evidence suggesting that consumers are, on average, more likely to take larger puff volumes when using a HTP compared to a classic cigarette. This observation is consistent with studies showing that HTPs lead to lower nicotine exposure (Bekki et al., 2017; Uchiyama et al., 2018; Vukas et al., 2023). Lower nicotine delivery could lead to compensatory puffing when product use does not sufficiently satisfy cravings (Vukas et al., 2023). Consumer use-behaviour and consumption data help ensure that modelling is reflective of real-world consumers. In this study, we assumed the same use-behaviour for cigarettes and HTPs users as the difference in nicotine levels between cigarettes and HTPs were not clear in all studies (Mallock et al., 2018; Salman et al., 2019; Rabenstein et al., 2023). Nicotine delivery and consumer satisfaction has also been shown to remain comparable between cigarette smoking and HTP use (Ogden et al., 2015; Picavet et al., 2016; Brossard et al., 2017; Roulet et al., 2019).

Public health experts worldwide have concluded that it is the toxicants in cigarette smoke generated by burning tobacco, and not the nicotine, which is the cause of smoking-related diseases. Therefore, whilst nicotine is a reliable biomarker for exposure to tobacco products, it does not provide any indication of health risks associated with smoking or the use of nicotine-containing products (Royal College of Physicians, 2016; FDA, 2022). Other biomarkers and health assessments are often used in conjunction to provide a more comprehensive understanding of the health impact of tobacco use. Both traditional cigarettes and HTP contain tobacco, but methods of consumption and associated health risk for these products differ. Cigarettes burn tobacco, producing smoke which contains numerous harmful chemicals with the number of smoke constituents being around 7,000 chemicals (Rodgman et al., 2000). Around 100 of these chemicals are classified by public health experts as causes or potential causes of smoking related disease (FDA, 2012). HTPs do not operate at temperatures high enough to burn tobacco. Since HTPs do not burn tobacco, they produce significantly fewer and lower levels of harmful chemicals compared to smoke from cigarettes (Schaller et al., 2016; Mallock et al., 2018; Salman et al., 2019; Vukas et al., 2023). Salman et al. (2019) for example, showed that reactive oxygen species (ROS) and carbonyl compound emissions were lower in HTP aerosol compared to cigarettes. Oxidative stress has been suggested as important part of several smoking-related diseases (Luettich et al., 2021).

*In vitro* experiments are essential to better understand differences in the potential effects of tobacco products. Several studies have demonstrated that measured reductions in toxicants in HTP aerosols compared to cigarette smoke can translate into reductions in *in vitro* toxicological effects (Schaller et al., 2016; Jaunky et al., 2018; Hattori et al., 2020; Dusautoir et al., 2021; Wang et al., 2021). Here, we investigated the effects of HTPs and the 1R6F Reference Cigarette, on the Phosphorylation of AP-1 transcription factor component, c-jun, which is a process involved in regulation of cellular stress responses such as cell cycling control and apoptosis (Dreij et al., 2010). Studies suggest that exposure to both HTP aerosol and cigarette smoke can lead to the activation of c-jun (Kogel et al., 2015; Chapman et al., 2023). However, the specific effects and the extent of c-jun activation may vary between these two forms of tobacco use. The elevated levels of c-jun observed in response to tobacco products’ exposure, make it a potentially useful biomarker for assessing tobacco-related effects on cellular processes.

In this work, to compare the effective concentrations of each product type, a study was designed to look at both the number of puffs delivered to cells (BEAS-2B) and the amount of nicotine delivered at the cell surface. The aim of this experiment was to determine the number of puffs required for the products to cause a minimum effective concentration for the c-jun cellular marker, measured using high content screening (HCS) and to relate the number of puffs with the delivery of nicotine to the cell surface. IVIVE was used to derive the exposure concentration (HEC) that matches the estimated *in vitro* effect (MEC) and then derive the equivalent plasma concentrations. Results show that it would be necessary to consume 3 HTP sticks at the same time to produce *in vivo* the effects seen *in vitro* under the conditions of this test. However, for the 1R6F cigarette, smoking only 1/6th of a stick would lead to the *in vivo* effect seen *in vitro* under the conditions of the test. Nicotine plasma concentrations ranged from 4 to 28 ng/mL after exposure to 1 stick of 1R6F at the MEC and at a nicotine concentration of a classic cigarette, respectively. For HTP, nicotine plasma concentrations range from 15.5 to 46.5 ng/mL after exposure to 1 stick of HTP at a nicotine concentration of a classic HTP stick and to 1 stick of HTP at the MEC, respectively. In comparison, plasma concentrations in clinical PK studies were between 14 and 20 ng/mL (Digard et al., 2013; Picavet et al., 2016; McEwan et al., 2019). At steady state, concentrations are higher, which is explained by the half-life of nicotine (1–3 h in blood). In the simulations with repeated use, sticks (1R6F and HTP) are consumed every hour for 10 h and therefore we can see an accumulation of nicotine in plasma over time until a steady state is achieved.

One of the modeling challenges in this study was that the *in vitro* testing was conducted with cigarettes and HTP smoke/aerosol which are complex mixtures including nicotine, organic acids, and carrier chemicals (propylene glycol and vegetable glycerol). The *in vitro* assays assessed the toxicity of the whole mixture, whereas the PBPK model and its parameterization was carried out for a specific compound, nicotine. Because of the various PK and pharmacodynamic properties of individual compounds in a mixture as well as the potential interactions between those chemicals, mixtures present a special challenge for conducting IVIVE (Hernandez and Tsatsakis, 2017). While important, these factors were not taken into consideration as the goal was to compare the potency of HTPs *versus* cigarettes. However, as a range of nicotine delivery products such as e-vapor products are gaining popularity among adult smokers, IVIVE modeling for mixtures must be considered (Zhang et al., 2021). Qualitatively, differences in irritant properties of the inhaled mixtures may be the primary cause of the c-jun responses being greater with cigarettes compared to the HTP. C-jun is an important marker as it plays a key role in cell cycle progression, that is achieved via the transcriptional repression of cell cycle inhibitors and the transcriptional activation of cell cycle progression machinery (Lukey et al., 2016).

Rodrigo et al. (2021) have suggested that the use of HTP may be associated with potentially reduced cancer and non-cancer endpoints based on their reduced harmful and potentially harmful constituent yields measured in aerosol when compared to cigarette smoke. Kusonic et al. (2023) stated that whilst HTP products have shown a reduced risk to human health when compared to the cigarettes, they still, however, contain compounds in the aerosol that can be detrimental to human health. The authors went on to state that there was not enough data obtained from independent studies indicating that the reduced amounts of toxic chemicals in the aerosol of HTP do not induce any harmful effects.

In conclusion, the results of this study demonstrate that for the 1R6F cigarette, consuming 1/6th of a stick would be required to induce the c-jun activation effects observed *in vitro*. Whereas, for HTP it would be necessary to consume 3 sticks simultaneously to produce *in vivo* the effects observed *in vitro*. This data further demonstrates the reduced potency of the HTP aerosol compared to cigarette smoke thereby adding to the weight of evidence that non-combustible next-generation products have the potential for reduced harm when compared to cigarettes. The QIVIVE approach demonstrates great promise in assisting human health risk assessments; however, further optimization and standardization is required to gain regulatory acceptance. Furthermore, biomarkers and health assessments other than nicotine and the measure of cellular stress also need to be studied to provide a more comprehensive understanding of the impact of tobacco use on an individual’s health.

## Data Availability

The raw data supporting the conclusion of this article will be made available by the authors, without undue reservation.
