# Supplementary material for: Use of quantitative in vitro to in vivo extrapolation (QIVIVE) for the assessment of non-combustible next-generation product aerosols
Source: Front Toxicol. 2024 Apr 11;6:1373325. doi: 10.3389/ftox.2024.1373325 (PMC11043521; doi:10.3389/ftox.2024.1373325)
Supplement: Supplementary file 1 [file DataSheet1.docx]

Supplementary Material

# Supplementary Data

**; Nicotine PBPK model created for Imperial Brand (July 2nd, 2020)**

METHOD RK4 ; Runge-Kutta 4th-order solver

STARTTIME = 0 ; Hours

STOPTIME = 24 ; Hours

DT = 0.001 ; Step Size

DTMAX = 0.0001 ; Controls the interval at which results are stored in memory

Minutes = TIME*60

Seconds = Minutes*60

; Nicotine molecular weight

MW = 162.23 ; g/mol

**; PHYSIOLOGICAL PARAMETERS**

BW = 81.2 ; Body Weight (kg)

; Blood flow rates

QC = 423.37 ; Cardiac output (L/hr)

QLc = 0.235 ; Fraction of blood flow to liver

QURTc = 0.00247 ; Fraction of blood flow to the upper respiratory tract (Campbell et al. 2015)

QTBc = 0.0075 ; Fraction of blood flow to the tracheo-bronchial region (Brown et al. 1997)

QALVc = 0.0067 ; Fraction of blood flow to the alveolar region (Butler et al. 1992)

TV = 1.3 ; Tidal Volume (L)

DS = 0.15 ; Dead space in the lung (L)

BR = 720 ; Breathing rate (/h)

; Scale blood flows by total cardiac output

QP = (TV-DS)*BR ; Alveolar flow (L/h)

QL = QLc*QC ; Liver blood flow (L/h)

QURT = QURTc*QC ; Upper respiratory tract blood flow (L/h)

QTB = QTBc*QC ; Tracheobronchial blood flow (L/h)

QALV = QALVc*QC ; Alveolar blood flow (L/h)

QRB = QC - QL - QURT - QTB - QALV ; Rest of the body blood flow (L/h)

Qbal = QL + QRB + QURT + QTB + QALV - QC ; Blood flow balance check =0?

; Surface area (cm2)

SAurt = 154.8 ; Upper airway

SAtb = 4440 ; Tracheobronchial region (Conducting airways with terminal bronchioles)

SAta = 6220 ; transitional (respiratory bronchioles)

SApulm = 700146.6 ; Pulmonary region (MMPD original value 706366.6 where was subtracted the respiratory bronchioles from Sarangapani to consider the difference in thickness in those 2 regions)

; Tissue thickness (cm) Sarangapani et al. 2005 and IRCP

TmucepithURT = 0.006 ; (mucus + epithelium) Nasal cavity

TmucepithTB =0.0066 ; Tracheobronchial region (IRCP)

TmucepithTA = 0.001 ; transitional airways (respiratory bronchioles)

TmucepithPULM = 0.0005 ; Pulmonary region

; The submucosal thickness was assumed to be approximately twice the epithelium thickness, based on histological sectioning (Matthew Bogdanffy, personal communication).

TURT = 0.012 ; submucosa nasal cavity

TTB = 0.0132 ; submucosa Tracheobronchial region

TTA = 0.002 ; submucosa Transitional airway

; Volumes lung region (L)

Vt1 = SAurt*TmucepithURT*0.001 ; Volume mucus + epithelium upper resp tract

Vt2 = SAtb*TmucepithTB*0.001 ; Volume mucus + epithelium TB region

Vt3 = SAta*TmucepithTA*0.001 + SApulm*TmucepithPULM*0.001 ; Volume mucus + epithelium pulmonary region (respiratory bronchioles transitional airways ta and alveoles pulm)

VURT = SAurt*TURT*0.001 ; *0.001 to adjust from cm^3 to L (1 cm^3 = 1 mL)

VTB = SAtb*TTB*0.001 ; Volume submucosa region

VPULM = SAta*TTA*0.001 ; Volume submucosa transitional airways (ta) as there is no submucosa region in the alveoli

VLu = VURT + VTB + VPULM + Vt1 + Vt2 + Vt3

VLuc = VLu / BW ; Lung fraction Brown has a value of 0.0073 as an average.

; Tissue Volume Fractions (L or kg per kg Body Weight)

VLc = 0.0197 ; Fraction of liver tissue

VArtc = 0.0142 ; Fraction of arterial blood (0.0569*1/4)

VVc = 0.0427 ; Fraction of venous blood (0.0569*3/4)

VRBc = 0.85-(VLc+VArtc+VVc+VLuc) ; Rest of the body volume

; Allometric scaling of tissue volumes (L)

VL = VLc*BW ; Liver volume

VArt = VArtc*BW ; Arterial blood volume

VV = VVc*BW ; Venous blood volume

VRB = VRBc * BW

VBal = 0.85 - VLc - VRBc - VArtc - VVc - VLuc ; Volume check (15% of the body is assumed not perfused) Clewell et al. 2004

**; BIOCHEMICAL PARAMETERS**

; Transfer coefficients

DLc = 4.87*10^-9 ; Nicotine diffusion coefficient for transfer across epithelium (cm2/s)

DL = DLc*3600 ; cm2/hr

; Units are cm3/h = mL/h (assuming density of 1 mL/cm3)

KtURT = (DL * SAurt) / ((TmucepithURT/2)+( TURT/2)) ; Upper respiratory tract (cm3/hr)

KtTB = (DL * SAtb) / ((TmucepithTB/2)+(TTB/2)) ; Tracheobronchial region (cm3/hr)

KtPULM = (DL * (SAta+SApulm)) / ((TmucepithPULM/2)+(TTA/2)) ; Pulmonary region (cm3/hr)

; Partition coefficients

PL = 7 ; liver/blood partition coefficient

PURT = 1.23 ; Upper respiratory tract partition coefficient

PTB = 0.1 ; Tracheobronchial region partition coefficient

PALV = 0.1 ; Alveolar region partition coefficient

PRB = 1.48 ; Rest of the body partition coefficient

; Protein Binding

Fu =0.95 ; plasma unbound fraction

; Renal and hepatic clearance

CLr = 4.25 ; Renal clearance (L/h)

CLint = 128.5 ; Hepatic Intrinsic Clearance (L/h)

**; Aerosol exposure**

; Particle/aerosol deposition fraction in respiratory tract regions from MPPD

FURT = 0.0082 ; Fraction deposited in the upper respiratory tract

FTB = 0.0608 ; Fraction deposited in the tracheobronchial region

FPULM = 0.1454 ; Fraction deposited in the pulmonary region

tBreath = 1/npuffpersession/60 ; time for one breath or puff (h)

Puff_dur = 2.3 ; Puff duration in second

puffduration = Puff_dur/3600 ; Puff duration in hour

Puff_int = 30 ; Puff interval in second

repeatpuff = Puff_int/3600 ; Puff interval in hour

npuffpersession = 10 ; Number of puffs per session

N_session = 1 ; Number of sessions

Npuff_total = Npuffpersession*N_session

is_inhtime = IF mod(TIME, repeatpuff) < puffduration THEN 1 ELSE 0

reachedMaxDosing = IF mod(TIME, 1)/repeatpuff >= npuffpersession THEN 1 ELSE 0

dosing = IF ( (is_inhtime = 1) AND (reachedMaxDosing = 0) AND (TIME < N_session) ) THEN 1 ELSE 0

DOSEnicotine_total = 0.56 ; Amount of nicotine (mg total)

DOSEnicotine_A = DOSEnicotine_total - (DOSEnicotine_total*Fvapor) ; Amount of nicotine as particles

Apuff = (DOSEnicotine_A/Npuff_total)*1000/MW ; Amount of nicotine particles in one puff transformed from mg to umol (umol)

Rpuff = Apuff/puffduration ; Rate of nicotine particles (umol/h)

**; Vapor exposure**

PuffVolume = 0.0695 ; Volume of 1 puff (L)

Fvapor = 0.2 ; Vapor fraction per puff (Haghnegahdar et al. 2018)

Cinh = (DOSEnicotine_total/(PuffVolume*Npuff_total))*1000*Fvapor /MW ; Concentration inhaled (umol/L) per puff

CI = Cinh*dosing

**; EQUATIONS**

; Liver compartment

AMTot' = CLint*Fu*CVL ; Rate of metabolism in the liver (umole/hr)

init AMTot = 0 ; Amount metabolized in the liver (umole)

AL' = QL * (Cart - CVL) - AMTot' ; Rate of change of nicotine in the liver (umole/hr)

init AL = 0 ; Amount of nicotine in the liver (umole)

CL = AL / VL ; Concentration of nicotine in the liver (umole/L)

CVL = CL / PL ; Free concentration leaving the liver (umole/L)

; Rest of the body compartment

ARB' = QRB * (Cart - CVRB) ; Rate of change of nicotine in the rest of the body (umole/hr)

init ARB = 0 ; Amount of nicotine in the rest of the body (umole)

CRB = ARB / VRB ; Concentration of nicotine in the rest of the body (umole/L)

CVRB = CRB / PRB ; Free concentration leaving the rest of the body (umole/L)

; Arterial blood

AMR' = CLr * Cart ; Rate of metabolism in blood (umole/hr)

init AMR = 0 ; Amount metabolized in blood (umole)

Aart' = QC * (CV - Cart) + (QP*CI) - AMR' ; rate of change in arterial blood (umole/hr)

init Aart = 0 ; Amount in arterial blood (umole)

Cart = Aart / VArt ; Concentration in arterial blood (umole/L)

; Venous blood

AV' = (QL * CVL) + (QRB * CVRB) + (QURT * CVURT) + (QTB * CVTB) + (QALV * CVALV) - (QC * CV) ; Rate of change in venous blood (umole/hr)

init AV = 0 ; Amount in venous blood (umole)

CV = AV / VV ; Concentration in venous blood (umole / L)

; Upper respiratory tract

ADepURT' = FURT*RPuff*dosing ; Rate of change of deposition in the URT (umol/hr)

init ADepURT = 0 ; Amount deposited in the URT (umol)

; Mucus + epith

At1' = ADepURT' - KtURT*0.001 * Ct1 / PURT ; Rate of change in the mucus (umole/hr)

init At1 = 0 ; Amount in the mucus (umole)

Ct1 = At1 / Vt1 ; Concentration (umole/L)

; Tissue

AURT' = KtURT *0.001* (Ct1 / PURT) + QURT * (Cart - CVURT) ; Rate of change in the URT (umole/hr)

init AURT = 0 ; Amount in the URT tissue (umole)

CURT = AURT / VURT ; Concentration in the URT tissue (umole/L)

CVURT = CURT / PURT ; Free concentration leaving the URT tissue (umole/L)

; TB region

ADepTB' = FTB * RPuff * dosing ; Rate of change of deposition in the TB region (umole/h)

init ADepTB = 0 ; Amount deposited in the TB (umol)

; Mucus + epith

At2' = ADepTB' - KtTB*0.001 * Ct2 / PTB ; Rate of change (umole/hr)

init At2 = 0 ; Amount (umole)

Ct2 = At2 / Vt2 ; Concentration (umole/L)

; Tissue

ATB' = KtTB*0.001 * (Ct2 / PTB) + QTB * (Cart - CVTB) ; Rate of change (umole/hr)

init ATB = 0 ; Amount (umole)

CTB = ATB / VTB ; Concentration (umole/L)

CVTB = CTB / PTB ; Free concentration leaving the tissue (umole/L)

; Pulmonary region

ADepPULM' = FPULM * RPuff * dosing ; Rate of change of deposition in the pulmonary region (umole/h)

init ADepPULM = 0 ; Amount deposited in the pulmonary region (umol)

; Mucus + epith

At3' = ADepPULM' - KtPULM*0.001 * Ct3 / PALV ; Rate of change (umole/hr)

init At3 = 0 ; Amount (umole)

Ct3 = At3 / Vt3 ; Concentration (umole/L)

; Tissue

APULM' = KtPULM*0.001 * (Ct3 / PALV) + QALV * (Cart - CVALV) ; Rate of change (umole/hr)

init APULM = 0 ; Amount of nicotine in the tissue (umole)

CALV = APULM / VPULM ; Concentration of nicotine in the tissue (umole/L)

CVALV = CALV / PALV ; Free concentration of nicotine leaving the tissue (umole/L)

; DOSE METRICS

CVmgpl = CV*MW ; Venous concentration in ug /L (ng/ml)

Cartmgpl = Cart*MW ; Arterial concentration in ug/L (ng/ml)

AUCCV' = CV ; Area under the curve (umol*h/L)

init AUCCV = 0

AUCmgpl = AUCcv * MW ; Area under the curve (ug*h / L)

; MASS BALANCE

InhDose' = QP*CI

init InhDose = 0 ; Amount inhaled (umole)

IN = ADepURT + ADepTB + ADepPULM + InhDose

STORED = AL + ARB + Aart + AURT + ATB + APULM + At1 + At2 + At3 + AV

Out = AMTot + AMR

MASSBAL = IN - STORED – Out

# Supplementary Figures and Tables

## Supplementary Tables

**Supplementary Table 1.** PBPK model parameters distributions

|  | CV | Distribution | SD |
| --- | --- | --- | --- |
| Puff duration | 0.3 | N | 0.69 |
| Puff interval | 0.3 | N | 9 |
| Breathing rate (BR) | 0.12 | LN | 86.4 |
| Tidal Volume (TV) | 0.12 | LN | 0.156 |
| BW | 0.22 | LN | 17.864 |
| Puff volume | 0.3 | N | 0.0208 |
| Fraction of vapor | 0.3 | Uniform | 0.06 |
| Partition coef. Rest of the body (PRB) | 0.3 | LN | 0.444 |
| Deposition fraction in pulm region (FPULM) | 0.3 | Uniform | 0.0437 |
| Cardiac Output | 0.17 | N | 71.97 |
| Dead Space (DS) | 0.12 | LN | 0.018 |
| Blood flow to the Liver (QLc) | 0.16 | N | 0.0376 |
| Hepatic Intrinsic Clearance (CLint) | 0.3 | LN | 38.55 |


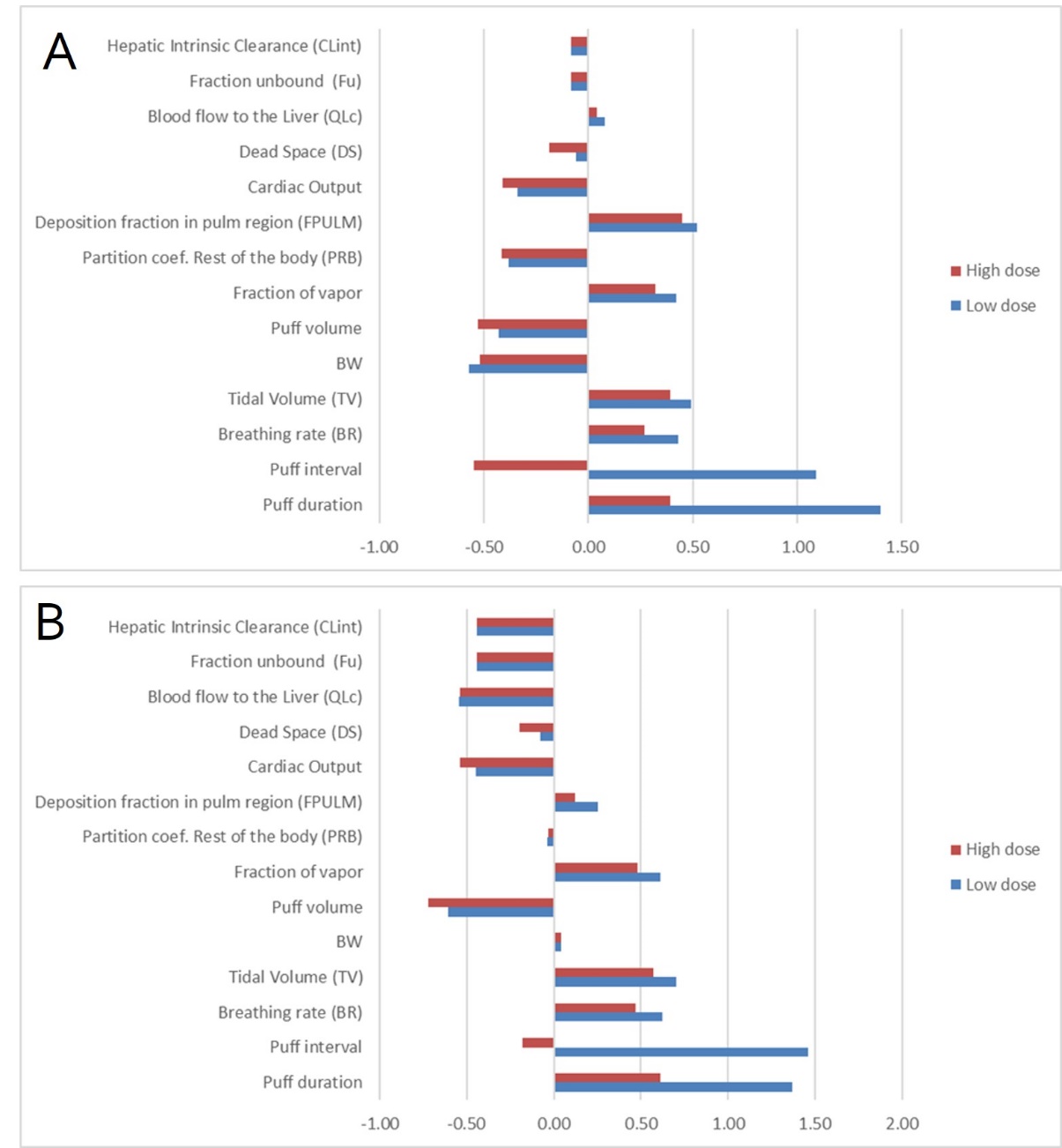


**Supplemental Figure 1**. Sensitivity analysis coefficient after inhalation evaluated for plasma Cmax (A) and plasma AUC (B).


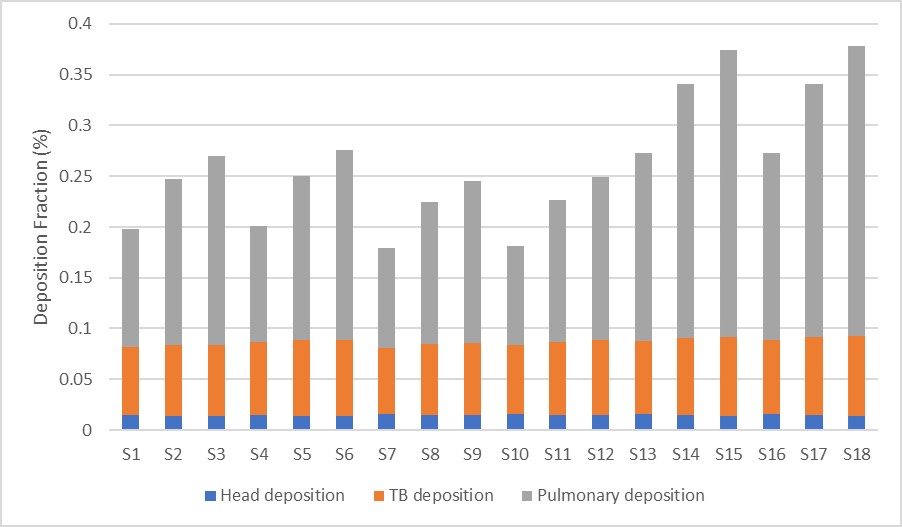


**Supplemental Figure 2**. Regional deposition fractions of inhaled aerosol particles as a function of different breathing scenarios (Table S2).

**Supplementary Table 2.** Breathing pattern scenario according to MPPD model.

| **Scenario*** | **Breathing rate (/h)** | **Tidal Volume (L)** | **Inspiration fraction** | **Pause fraction** | **Inhalation time (s)** | **Exhalation time (s)** | **Notes** |
| --- | --- | --- | --- | --- | --- | --- | --- |
| 1 | 12 | 1.3 | 0.5 | 0 | 2.5 | 2.5 | Deep Breathing at resting, same inh/Exh time, no breath hold |
| 2 | 12 | 1.3 | 0.5 | 0.1 | 2.5 | 2 | Deep Breathing at resting, same inh/exh time, short breath hold |
| 3 | 12 | 1.3 | 0.5 | 0.25 | 2.5 | 1.25 | Deep Breathing at resting, same inh/exh time, extended breath hold |
| 4 | 12 | 1.3 | 0.3 | 0 | 1.5 | 3.5 | Deep Breathing at resting, shorter inhalation and extended exhalation, no breath hold |
| 5 | 12 | 1.3 | 0.3 | 0.1 | 1.5 | 3 | Deep Breathing at resting, shorter inhalation and extended exhalation, short breath hold |
| 6 | 12 | 1.3 | 0.3 | 0.25 | 1.5 | 2.25 | Deep Breathing at resting, shorter inhalation and extended exhalation, extended breath hold |
| 7 | 12 | 0.75 | 0.5 | 0 | 2.5 | 2.5 | Normal Breathing at resting, same inh/Exh time, no breath hold |
| 8 | 12 | 0.75 | 0.5 | 0.1 | 2.5 | 2 | Normal Breathing at resting, same inh/exh time, short breath hold |
| 9 | 12 | 0.75 | 0.5 | 0.25 | 2.5 | 1.25 | Normal Breathing at resting, same inh/exh time, extended breath hold |
| 10 | 12 | 0.75 | 0.3 | 0 | 1.5 | 3.5 | Normal Breathing at resting, shorter inhalation and extended exhalation, no breath hold |
| 11 | 12 | 0.75 | 0.3 | 0.1 | 1.5 | 3 | Normal Breathing at resting, shorter inhalation and extended exhalation, short breath hold |
| 12 | 12 | 0.75 | 0.3 | 0.25 | 1.5 | 2.25 | Normal Breathing at resting, shorter inhalation and extended exhalation, extended breath hold |
| 13 | 5 | 1.3 | 0.5 | 0 | 6 | 6 | Deep slow breathing, same inh/Exh time, no breath hold |
| 14 | 5 | 1.3 | 0.5 | 0.1 | 6 | 4.8 | Deep slow breathing, same inh/exh time, short breath hold |
| 15 | 5 | 1.3 | 0.5 | 0.25 | 6 | 3 | Deep slow breathing, same inh/exh time, extended breath hold |
| 16 | 5 | 1.3 | 0.3 | 0 | 3.6 | 8.4 | Deep slow breathing, shorter inhalation and extended exhalation, no breath hold |
| 17 | 5 | 1.3 | 0.3 | 0.1 | 3.6 | 7.2 | Deep slow breathing, shorter inhalation and extended exhalation, short breath hold |
| 18 | 5 | 1.3 | 0.3 | 0.25 | 3.6 | 5.4 | Deep slow breathing, shorter inhalation and extended exhalation, extended breath hold |

*Particle diameter CMD = 0.163 µm with GSD 1.44
